# Cancer-testis antigens MAGEA proteins are incorporated into extracellular vesicles released by cells

**DOI:** 10.18632/oncotarget.26979

**Published:** 2019-06-04

**Authors:** Anneli Kuldkepp, Magda Karakai, Eve Toomsoo, Olavi Reinsalu, Reet Kurg

**Affiliations:** ^1^ Institute of Technology, University of Tartu, Tartu, Estonia

**Keywords:** cancer-testis antigens, MAGEA, extracellular vesicles, microvesicles

## Abstract

Melanoma-associated antigen A (MAGEA) family proteins represent a class of tumor antigens that are expressed in a variety of malignant tumors, but their expression in normal tissues is restricted to germ cells. MAGEA family consists of eleven proteins that are highly conserved sharing the common MAGE homology domain (MHD). In the current study, we show that MAGEA4 and MAGEA10 proteins are incorporated into extracellular vesicles released by mouse fibroblast and human osteosarcoma U2OS cells and are expressed, at least partly, on the surface of released EVs. The C-terminal part of the protein containing MHD domain is required for this activity. Expression of MAGEA proteins induced the budding of cells and formation of extracellular vesicles with 150 to 1500 nm in diameter. Our data suggest that the release of MAGEA-positive EVs is at least to some extent induced by the expression of MAGEA proteins itself. This may be one of the mechanisms of MAGEA proteins to induce cancer formation and progression.

## INTRODUCTION

Cancer testis antigens (CTAs) are a large family of tumor-associated antigens that are normally expressed only in the human germ line, but aberrant expression is detected in various human tumors. MAGEA (melanoma associated antigen A) subfamily proteins were the first tumor associated antigens identified at the molecular level [1]. They are recognized by cytotoxic T lymphocytes and evoke a strong T cell reactivity against autologous tumor cells in culture [2]. *MAGEA* is a sub-family of 12 genes (*MAGEA1* to *-A12*) located in the q28 region of the X chromosome [3]. Members of the MAGEA subfamily proteins are normally expressed only in testis or placenta and their restricted expression suggests that they may function in germ cell development. MAGEA proteins were also detected in the early development of the central nervous system and the spinal cord and brainstem of peripheral nerves, revealing that MAGEA proteins may also be involved in neuronal development [4]. The aberrant expression is detected in tumor cells of multiple types of human cancer [5–7] probably due to genome-wide epigenetic reprogramming taking place in tumor cells [8, 9]. MAGEA expression is observed mainly in cancers that have acquired malignant phenotypes, invasiveness and metastasis, and the expression of MAGEA family proteins has been linked to poor prognosis in cancer patients [10, 11].

All MAGE proteins share the common MAGE homology domain (MHD), a highly conserved domain consisting of approximately 170 amino acids. Within the MAGEA family, the MHD encompasses up to 70% of the protein, the areas flanking the MHD region vary between sub-families and therefore may determine their specific functions. The MHDs within MAGEA sub-family members are 60–80% conserved [12], however, despite the sequence and structural similarities, the MAGEAs are structurally dynamic proteins [13], which may undergo conformational changes that allow for interaction with distinct protein domains, thereby conferring unique functions to individual MAGEs [12].

The biological functions and underlying regulatory mechanism of MAGEA proteins expression in cancer is still not fully understood. Different studies have associated MAGEA proteins with pro-tumorigenic activities such as dysregulation of p53 [14–16], involvement in fibronectin-controlled cancer progression [17] and both pro- and anti-apoptotic activities of different cells [18–20]. Multiple MAGE family proteins bind E3 RING ubiquitin ligases, for instance the MAGEA3/6 proteins bind TRIM28/Kap1 and enhance its ubiquitin ligase activity [21, 22], which allows to classify them as MAGE family of ubiquitin ligases [12]. Deletion of six members of the *Magea* gene cluster in mouse model showed that these genes are crucial in maintaining normal testicular size and protect germ cells from excessive apoptosis under genotoxic stress [23].

Extracellular vesicles (EVs) are circulating membrane vesicles released into the extracellular space by all types of cells. The umbrella term ‘extracellular vesicle’ includes exosomes, microvesicles and apoptotic bodies, traditionally distinguished by their size and biogenesis [24]. Exosomes are complex 20 - 100 nm vesicles formed by the inward budding of endosomal membranes to form large multivesicular bodies (MVBs). These vesicles are released extracellularly when MVBs fuse with the plasma membrane. By contrast, microvesicles (100 nm–1 μm) and apoptotic bodies (1–5 μm) result from the direct outward budding and fission of the plasma membrane. EVs carry a cargo of soluble and membrane-bound protein, lipids, metabolites, DNA, and RNA (mRNA, miRNAs, and other small regulatory RNAs), contained within a protective lipid bilayer [24]. Cells can communicate with neighboring or distant cells through EVs; EVs can enter a target cell via receptor-ligand interactions, direct binding to the plasma membrane, phagocytosis and micropinocytosis, or clathrin-mediated endocytosis. EVs contain the content of its origin cells; through delivering the cargo they can modify the physiological state of the recipient cell [25–27].

We recently showed that MAGE4 and MAGEA10 proteins are efficiently incorporated into retrovirus Gag protein induced virus-like particles (VLPs) [28]. In the current study, we show that MAGEA4 and MAGEA10 proteins are incorporated into naturally occurring EVs released by mouse fibroblast and human osteosarcoma U2OS cells and are expressed on the surface of EVs. Expression of MAGEA proteins induced the budding of cells and formation of extracellular vesicles with different sizes, 150 to 1500 nm in diameter. Our data suggest that at least some part of MAGEA proteins expressed in cells is attached to the outer surface of EVs, probably to microvesicles and/or small apoptotic bodies, which may have a role in cancer progression.

## RESULTS

### MAGEA proteins are incorporated into EVs

**Figure 1:**
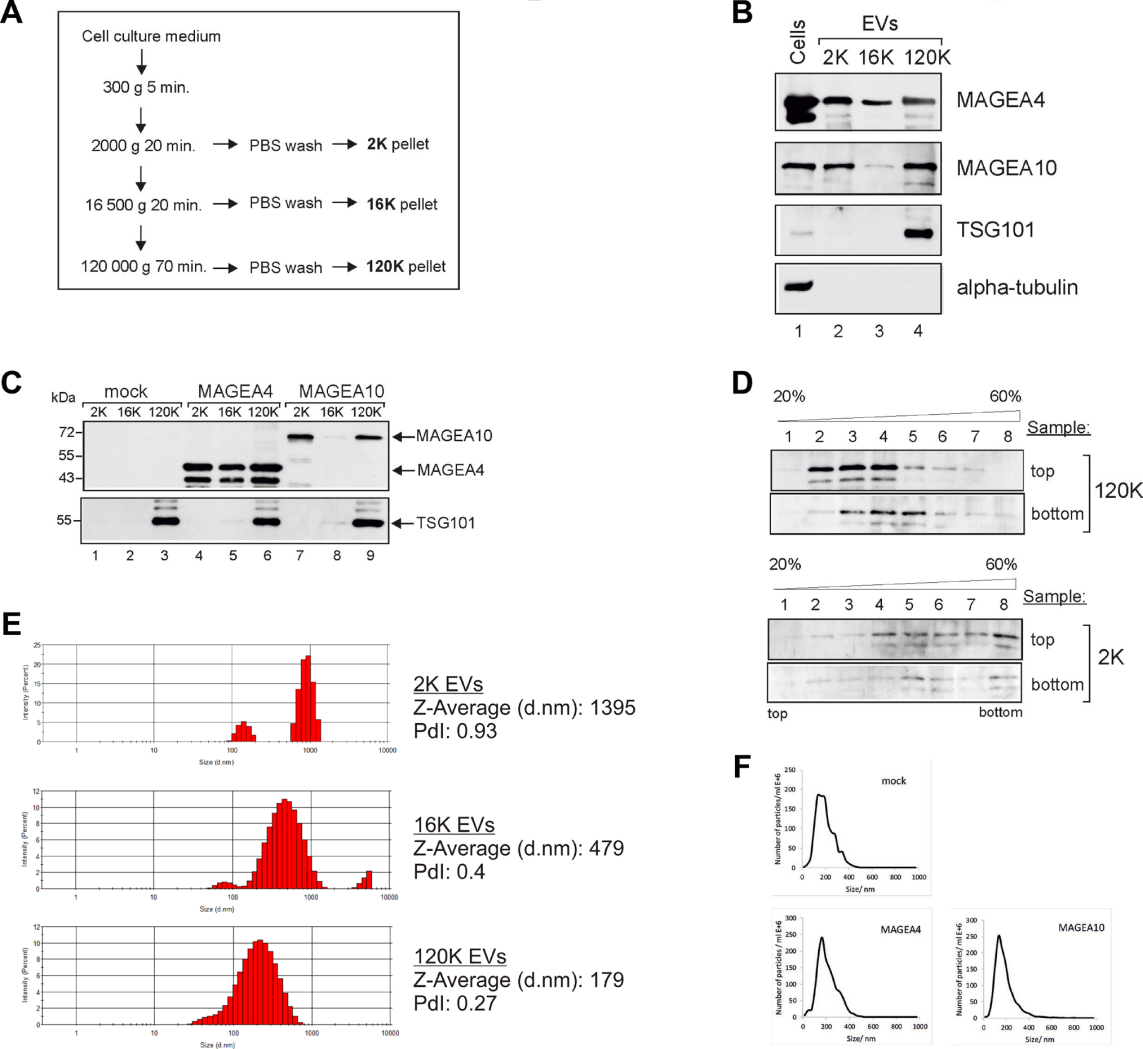
MAGEA proteins are incorporated into EVs. (**A**) Schematic representation of the purification scheme of EVs. (**B**) Western blot analysis of COP5 cells and EVs purified from COP5 cells ectopically expressing recombinant MAGEA proteins. The presence of proteins was analysed by specific antibodies against MAGEA4, MAGEA10, TSG101 (T5701; Sigma-Aldrich) and alpha-tubulin (T5168; Sigma-Aldrich). 5% of each EV fraction was loaded to the gel. (**C**) Western blot analysis of EVs purified from COP5 cells ectopically expressing recombinant MAGEA proteins using anti-E2Tag antibody (for MAGEA4 and MAGEA10) and anti-TSG101 antibody (T5701; Sigma-Aldrich). 5% of each EV fraction was loaded to the gel. (**D**) Western blot analysis of EVs obtained with ultracentrifugation through stepwise sucrose density gradient (20%, 35%, 45%, 60%) at 120 000 g for 1.5 hours at 4^°^ C. Gradient was divided into 8 fractions and the presence of MAGEA4 protein in each fraction was analyzed with MAGEA4 specific antibody. Top and bottom on the right size of the image depicts the loading place of samples. (**E**) Physical characterization of EVs as assessed by DLS (Dynamic Light Scattering). The hydrodynamic diameter (Z-average) and polydispersity index (PdI) are shown on the right. (**F**) Physical characterization of 120K EVs as assessed by NTA (Nanoparticle tracking analysis).

To analyze the expression of MAGEA proteins in extracellular vesicles, we purified EVs from mouse fibroblast cells expressing recombinant MAGEA4 and MAGEA10 proteins using differential centrifugation as described in [29]. Briefly, the cell culture media depleted from FCS-derived EVs was collected 72 hours after transfection and subjected to centrifugation as shown in Figure 1A. All the pellets were washed with PBS and analyzed by Western blotting using antibodies against MAGEA proteins, exosome marker TSG101 and tubulin. MAGEA4 positive EVs were detected in all fractions while MAGEA10 signal was mostly in 2K and 120K pellets (Figure 1B). TSG101 was detected only in 120K pellet fractions confirming that this fraction contains exosomes. We also measured the amount of the total protein in our EV samples by Bradford assay. On average, we obtained approximately 35 μg of 2K, 10 μg of 16K and 50 μg of 120K vesicles from 4 ×10^7^ of transfected mouse fibroblast cells. We have added the E2Tag epitope tag [30] to the C-terminus of MAGEA proteins which allows us to compare the amount of MAGEA4 and MAGEA10 positive EVs (Figure 1C). The amount of EVs varied between different experiments, but we usually detected more MAGEA4 EVs than MAGEA10 EVs. In the case of MAGEA4, EVs contained also smaller fragments of the protein (Figure 1C).

Next we used the sucrose gradient to sub-fractionate the 2K and 120K pellets. Density gradients are a classic method to separate membrane-enclosed vesicles according to their size, flotation speed and equilibrium density. The 2K and 120K pellets were loaded either to the top or bottom of the stepwise density gradient and the fractions were collected after ultracentrifugation. The membrane-enclosed vesicles loaded on the bottom of the gradient float upwards, whereas protein aggregates remain in the bottom of the tube. In the case of 120K pellet, the MAGEA4 EVs were detected in fractions 3–5 when the probe was loaded to the bottom, and in fractions 2–4 when the probe was loaded on the top of the gradient (Figure 1D). In contrast, the material of 2K fraction was distributed all over the gradient in both cases, regardless of whether the EVs were loaded onto the top or the bottom of the gradient. This is consistent with the results of DLS analysis (Figure 1E) which showed the polydispersity index PdI 0.27 for 120K EVs and 0.93 for 2K EVs, suggesting that 120K EVs are relatively homogenous fraction containing mostly small EVs, while 2K pellet contained different EVs, both small and big EVs. The 120K EVs were also analyzed by NTA to show their size distribution profiles: EVs isolated from mock cells peaked at 146 nm, MAGEA4 EVs peaked at 172 nm and MAGEA10 EV at 153 nm (Figure 1F). These data suggest that MAGE proteins are incorporated into EVs of different sizes, both small and big vesicles released by mouse fibroblast cells expressing recombinant MAGEA proteins.

### MAGEA proteins are expressed on the surface of EVs

**Figure 2:**
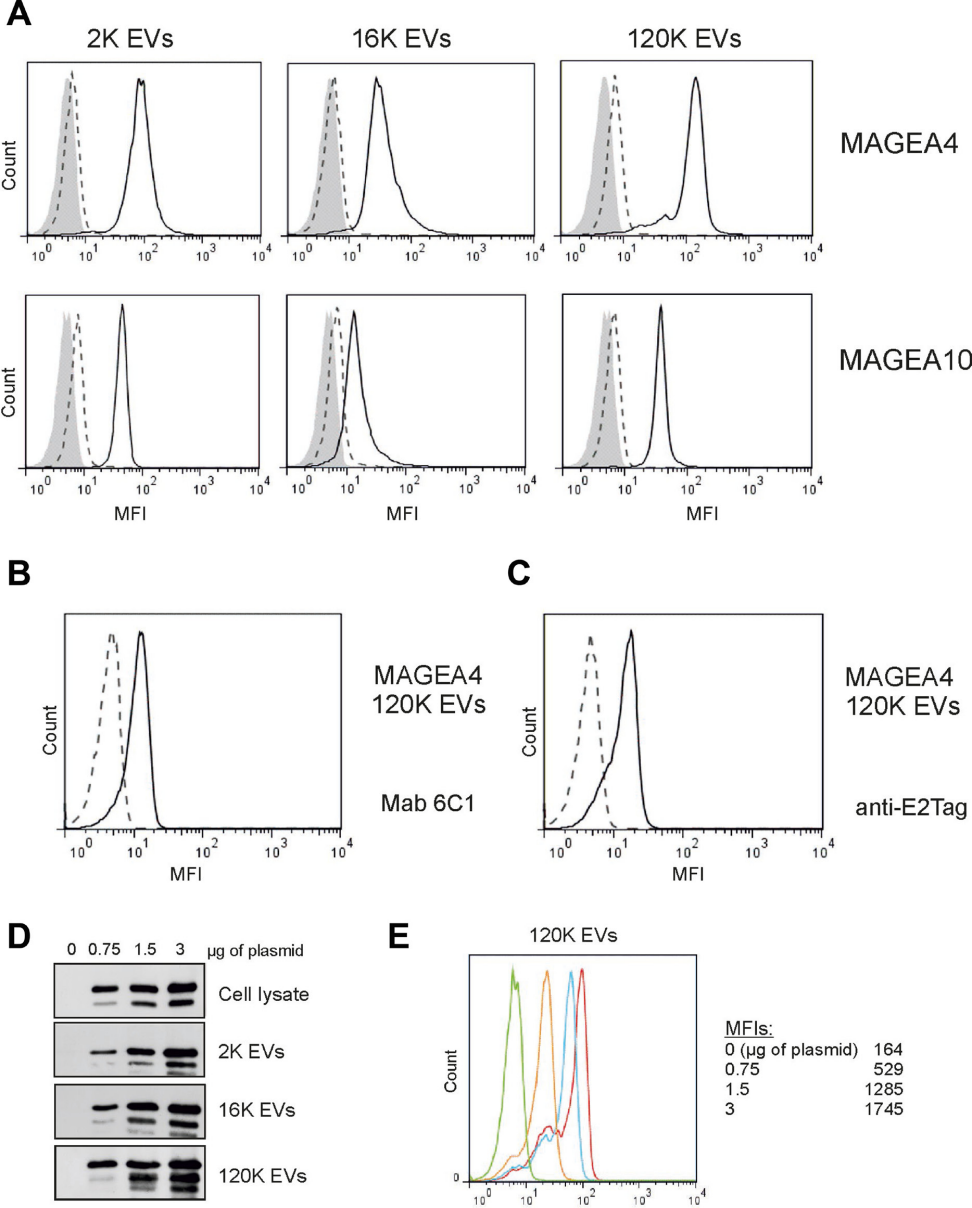
MAGEA proteins are expressed on the surface of EVs. (**A**) Flow cytometer analysis of EVs bound to aldehyde/sulfate latex beads as described in the Materials and Methods section. EVs were incubated with the MAGEA4 and MAGEA10 specific antibodies, respectively. The solid lines correspond to EVs containing recombinant MAGEA proteins, the dotted lines to EVs without recombinant protein and grey area shows the signal obtained with secondary Alexa488-labelled antibody. One representative experiment out of the three performed is shown. MFI = mean fluorescence intensity. (**B**, **C**) FACS analysis of 120K EVs bound to aldehyde/sulfate latex incubated with Mab 6C1 and anti-E2Tag antibody, respectively. (**D**) Western blot analysis of cells and EVs obtained after transfection of COP5 cells with different concentrations of MAGEA4 expression plasmids. Analysis was performed with MAGEA4 specific antibodies. 5% of each EV fraction was loaded to the gel. (**E**) 120K EVs shown in panel (**D**) were bound to aldehyde/sulfate latex beads and incubated with MAGEA4 specific antibodies. Red line shows results of EVs obtained with 3 μg of expression plasmid (per 5 × 10^6^ cells), blue corresponds to 1.5 μg, yellow to 0.75 μg and green is a mock control (no expression).

In order to determine whether MAGEA proteins are expressed on the surface of EVs, we performed the immunostaining analysis of EVs bound to aldehyde/sulfate latex beads [28]. As shown in Figure 2A, MAGEA4 and MAGEA10 specific antibodies recognized all three subtypes of EVs by flow cytometer analysis suggesting that at least part of these proteins are exposed on the surface of EVs. High fluorescence signals (mean fluorescence intensity; MFI) were detected in case of both 2K and 120K EVs and lower MFIs in 16K EVs. For MAGEA4 EVs, the MFI was 2570 for 2K and 3383 for 120K and in case of MAGEA10 EVs, 1191 for 2K and 1049 for 120K EVs, while the MFIs for mock control were 160 for 120K and 170 for 120K EVs (Figure 2A). MAGEA4 surface expression of 120K EVs was also tested with two other antibodies, with mouse monoclonal antibody 6C1, which recognizes all members of the MAGEA subfamily [31], and with antibody against E2Tag fused to the C-terminus of the protein. The surface expression of MAGEA4 protein was detected with both antibodies (Figure 2B, 2C) showing independently that the MAGEA4 protein is expressed on the surface of EVs.

Next we transfected the cells with increasing concentrations of MAGEA4 expressing plasmids and isolated the EVs as depicted in Figure 1A. The increase of the MAGEA4 protein signal in all EVs was detected (Figure 2D). FACS analysis of EVs showed that the amount of the protein exposed on the surface of EVs was also increased (Figure 2E). These data suggest that relatively large amounts of MAGEA4 protein can be incorporated into membranous vesicles of different sizes.

**Figure 3:**
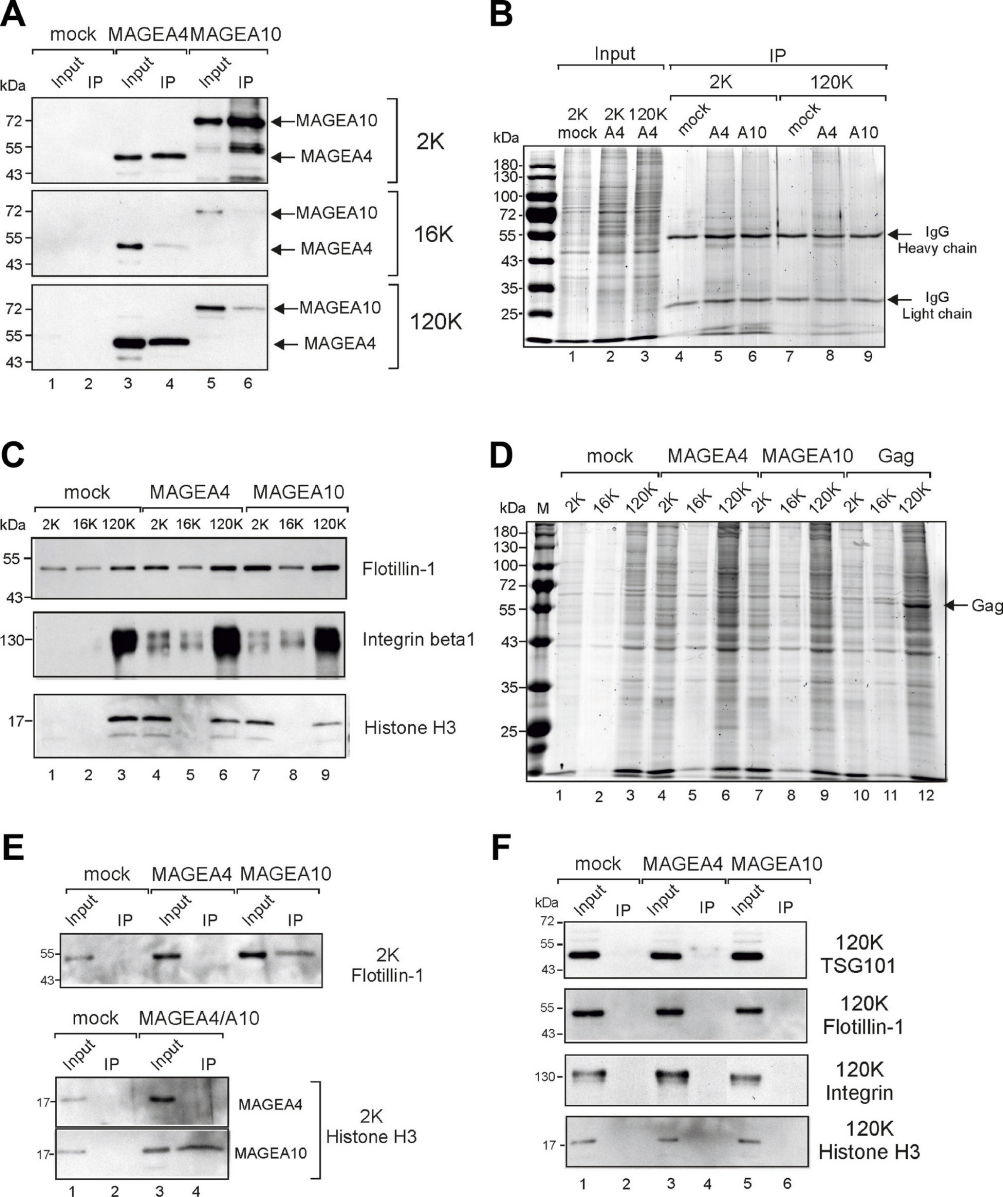
Immunoprecipitation of MAGEA-EVs. (**A**) MAGEA4 and MAGEA10 EVs were pulled down from 2K, 16K and 120K fractions using MAGEA4 and MAGEA10-specific antibodies. Immunoprecipitation was performed as depicted in Materials and Methods section and Western blot analysis was performed with anti-E2Tag antibody. Input (10%) and IP are shown. (**B**) Coomassie Blue staining of SDS-PAGE of immunoprecipitated material of panel (**A**). The positions of antibody heavy and light chains are shown by arrows. (**C**) Western blot analysis of EVs purified from COP5 cells ectopically expressing recombinant MAGEA proteins using flotillin, (610820; BD Transduction Laboratories), anti-integrin beta 1 (ab179471; Abcam) and anti-histone H3 (ab1791; Abcam) antibodies. 5% of each EV fraction was loaded to the gel. (**D**) Coomassie Blue staining of SDS-PAGE of 2K, 16K and 120K fractions isolated from COP5 cells expressing MAGEA4, MAGEA10 and MLVGag proteins, respectively. The position of MLVGag protein in VLPs is shown by arrow. (**E**) Analysis of immunoprecipitated material from 2K fractions with anti-flotillin and anti-histone H3 antibodies. (**F**) Analysis of immunoprecipitated material from 120K fractions with anti-TSG101, anti-flotillin, anti-integrin beta 1 and anti-histone H3 antibodies.

To confirm the surface expression of MAGEA proteins, we performed the immunoprecipitation of MAGEA-specific EVs. As shown in Figure 3A, we were able to immunoprecipitate both MAGEA4 and MAGEA10-specific EVs, however, with different efficiencies. We pulled down MAGEA4-EVs from 2K and 120K fractions with the same efficiencies while MAGEA10-EVs were recovered mostly from the 2K fraction (Figure 3A). In both cases, 16K fraction contained very few MAGEA-specific EVs in IP fraction. Similar results were obtained by immunoprecipitating EVs with both MAGEA-specific and E2Tag antibodies as the epitope tag is efficiently exposed onto the surface of EVs previously shown by FACS (Figure 2C). These results may reflect the different amount of MAGEA proteins bound to the surface of EVs or alternatively, the different origin of vesicles. The analysis of 2K and 120K IP fractions by staining of the SDS-gel with Coomassie Blue showed a lot of proteins in IP material (Figure 3B, lanes 5, 6, 8) suggesting that we pulled down vesicles rather than proteins itself. In order to analyze more EV markers in addition to TSG101, we included flotillin, shown to be enriched in 2K and 120K fraction [29], and integrin, enriched in 120K, into our study (Figure 3C). The total protein content and distribution between different fractions is shown in Figure 3D. In addition, histone H3 was included to test the hypothesis that EVs represent small apoptotic bodies. As shown in Figure 3C, histone was enriched in 2K fractions of MAGEA-EVs (compare lane 1 with lanes 4 and 7), and we were able to detect H3 in MAGEA10-specific IP fractions of 2K material (Figure 3E). Flotillin was detected in the same fraction (Figure 3E). This suggests that the 2K fraction of MAGEA10 contains at least to some extent small apoptotic bodies, but also microvesicles originating from the cell. At the same time, we could not detect the exosome marker TSG101, flotillin nor integrin in pulled down material of 120K EVs (Figure 3F).

### The C-terminal part of MAGE-A4 is required for incorporation into EVs

**Figure 4:**
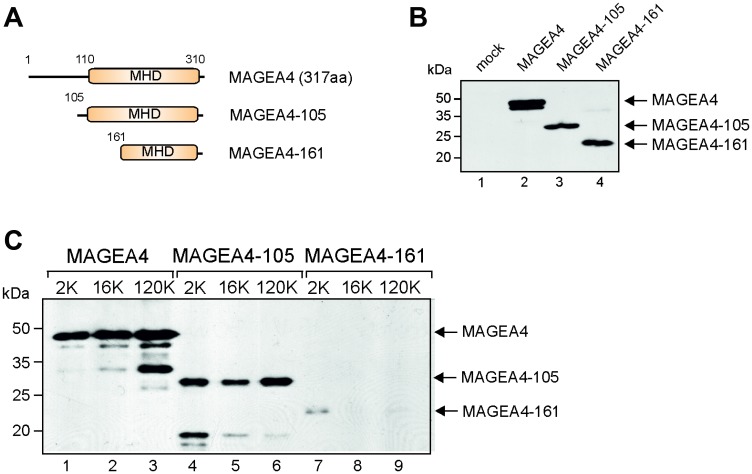
The C-terminal part of MAGEA4 is required for incorporation into EVs. (**A**) Schematic representation of MAGEA4 deletion mutants. Numbers depict the positions of amino acids. (**B**) Western blot analysis of cell lysates expressing MAGEA4 deletion mutants using anti-E2Tag antibody. (**C**) Western blot analysis of EVs isolated from cell culture supernatant expressing MAGEA4 deletion mutants using anti-E2Tag antibody.

FACS analysis suggested that the C-terminus of the MAGEA4 protein is exposed to the surface of EVs as MAGEA4 EVs containing epitope tag in its C-terminus was readily detected by E2Tag-specific antibodies (Figure 2C). Next we made two truncated proteins, MAGEA4-105 and MAGEA4-161 (Figure 4A), where the N-terminal 104 and 160 amino acids, respectively, were deleted. MAGEA4-105 keeps the entire MHD domain, but MAGEA4-161 disrupts the conserved MHD domain; both truncated proteins contain the E2Tag in the C-terminus. We analyzed these proteins similarly to the full-length protein, conducting Western blot analyses with the antibody recognizing the epitope tag. As shown on Figure 4B, both truncated proteins were expressed in the cells. Analysis of EVs isolated from culture media of transfected cells revealed that MAGEA4-105 was detected in EVs, but MAGEA4-161was not (Figure 4C). This confirms that the C-terminal part of the MAGEA4 protein is required for its incorporation into EVs and that the entire MHD domain is required for this activity.

### Endogenously expressed MAGEA4 is incorporated into EVs

**Figure 5:**
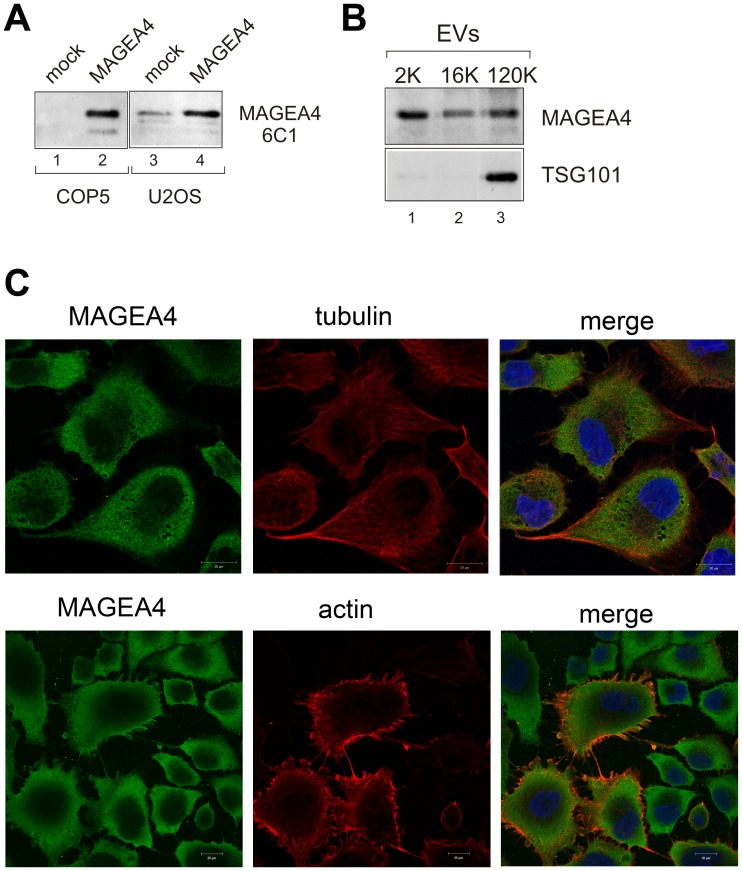
Endogenous MAGEA4 is incorporated into EVs. (**A**) Western blot analysis of COP5 and U2OS cells with 6C1 antibody (sc-20034; Santa Cruz). Ectopically expressed MAGEA4 protein is shown on lanes 2 and 4. (**B**) Western blot analysis of EVs isolated from the supernatant of U2OS cells using MAGEA4 and anti-TSG101 antibodies. (**C**) The indirect immunofluorescence analysis U2OS cells with MAGEA4, anti-tubulin and anti-actin antibodies. The Alexa-488 (MAGEA4) and Alexa-568 (tubulin and actin) conjugated antibodies were used as secondary antibodies.

All the experiments shown so far were done with EVs purified from transfected mouse fibroblast COP5 cells ectopically expressing recombinant MAGEA proteins. Most of the cell lines do not express MAGEA proteins, one of the few exceptions is human osteosarcoma cell line U2OS, which expresses several MAGEA proteins including MAGEA4 and to lesser extent MAGEA10 [15, 32]. The expression of MAGEA4 in U2OS cells was verified by Western blot analysis using MAGEA specific antibody 6C1 (Figure 5A). In order to test whether the endogenous MAGEA4 protein was also incorporated into EVs, we isolated EVs from U2OS cells and analyzed them with MAGEA4-specific antibodies. As shown in Figure 5B, the MAGEA4 protein is incorporated into EVs released by U2OS cells.

In U2OS cells, we can also follow the localization of the endogenous MAGEA4 protein. Immunofluorescence analysis of U2OS cells with MAGEA4 specific antibody showed cytoplasmic localization (Figure 5C), which is consistent with our previous results with recombinant protein [28]. In some cells, MAGEA4 localization to the membrane filaments was detected. Co-staining with antibodies against tubulin did not show any co-localization, while co-staining with beta-actin antibodies showed some co-localization of MAGEA4 and actin on the edges of the cell and filaments. However, this seemed to be random and was detected only in some cells. Interestingly, only few cells were positive for beta-actin, the others were not. We also saw tiny dots between the cells which may refer to EVs released into the extracellular space (Figure 5C).

### MAGEA proteins induce the budding and shedding of EVs from cells and formation of vesicles with different sizes

**Figure 6:**
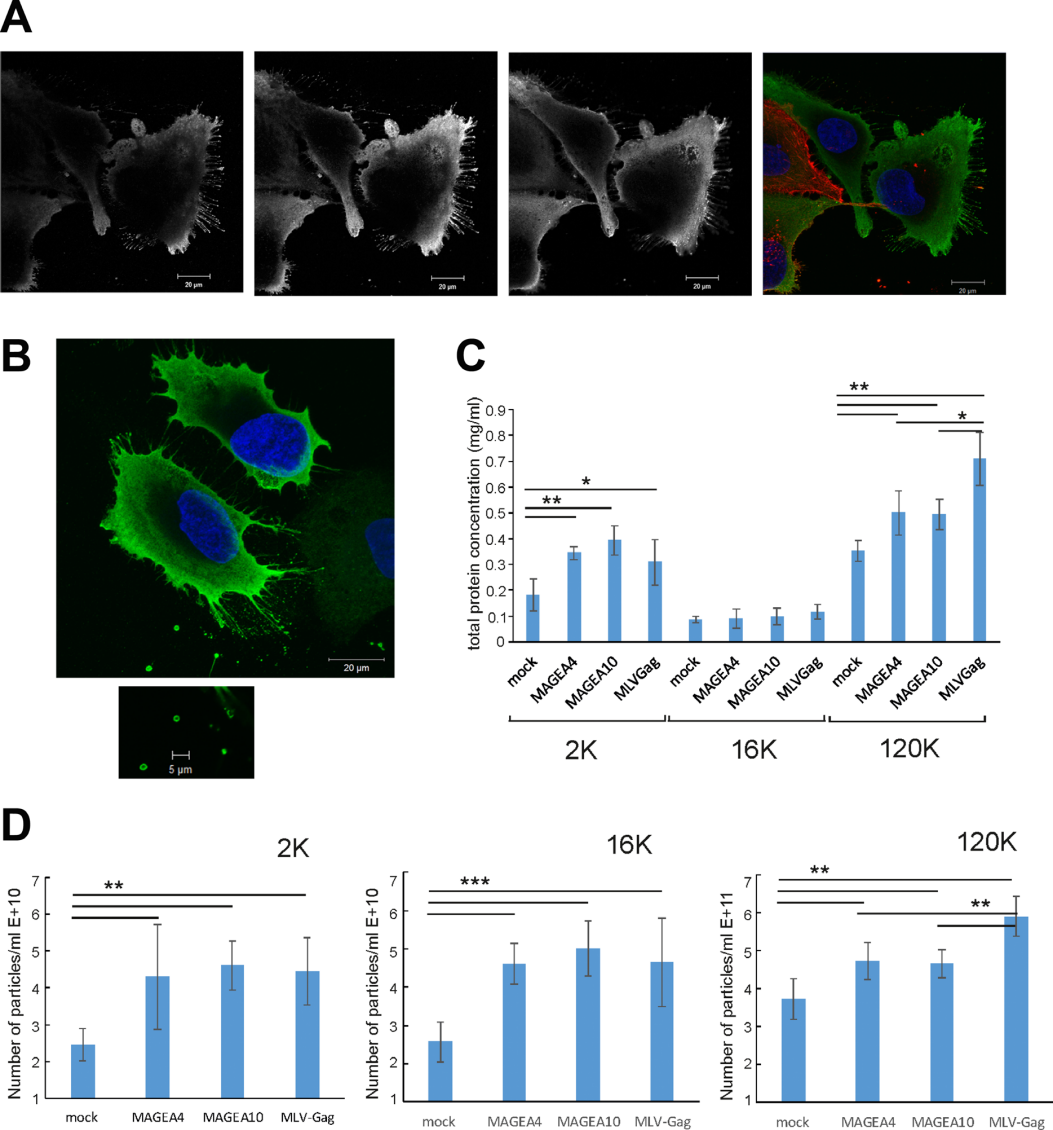
MAGEA proteins induce shedding and budding of EVs from cells. (**A**, **B**) MAGEA4 protein was overexpressed in U2OS cells and the cells were analysed by indirect immunofluorescence microscopy using MAGEA4 (green) and beta-actin (red) antibodies. Three Z-stack images (grey) and the merge picture is shown. DAPI was used to stain the nucleus. (**C**) The amount of total protein of EVs isolated from COP5 cells transfected with MAGEA and MLVGag proteins. Average of five experiments with standard deviations is shown. The *p*-value is below 0.05 (^*^) or below 0.01 (^**^) using Students paired *t*-test with a two-tailed distribution. (**D**) The amount of EVs isolated from COP5 cells transfected with MAGEA and MLVGag proteins analyzed by NTA. Average of eight measurements from four different experiments with standard deviations is shown. The *p*-value is below 0.005 (^**^) or below 0.001 (^***^) using Students paired *t*-test with a two-tailed distribution.

Over-expression of MAGEA4 protein in U2OS cells induced the formation of filaments budding from the cells (Figure 6A). The filaments were positive for MAGEA4 protein, but negative for beta-actin staining. In some cases, we could also follow the EVs with 1 to 2 μm in diameter which had already budded from the cells (Figure 6B). To find out whether the expression of MAGEA proteins induces budding or shedding of cells, we measured the amount of total protein after purification of vesicles in all EV fractions. In this experiment, MLVGag was used as a positive control as the retrovirus Gag protein induces the budding of cells and formation of VLPs [28] (Figure 3D). As shown in Figure 6C, both MAGEA4 and MAGEA10 proteins induced the increase of total protein in 2K as well as 120K fractions of EVs isolated from COP5 cells in a statistically significant manner (Figure 6C). The number of vesicles isolated from mouse fibroblasts was also analyzed by NTA (Figure 6D), which confirmed that the expression of MAGEA4 and MAGEA10 proteins enhanced the formation of EVs of different sizes.

**Figure 7:**
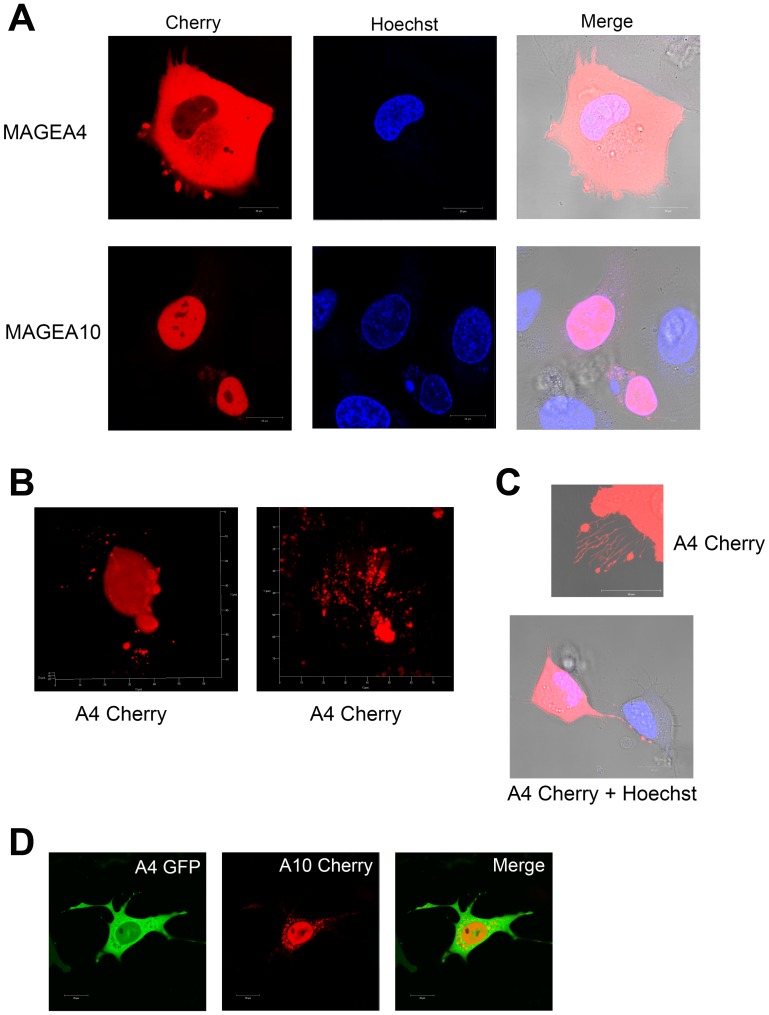
Live-cell imaging of U2OS cells overexpressing MAGEA proteins. (**A**) MAGEA4-Cherry and MAGEA10-Cherry proteins were electroporated into U2OS cells and analysed 48 hours post-transfection. Hoechst 33342 was added 15 minutes before microscope analysis to label the nuclei. Analysis was performed using confocal laser scanning microscope LSM710 (Zeiss). (**B**) Z-stacks were made from MAGEA4-expressing cells spreading EVs and 3D image was created by ZEN software. (**C**) MAGEA4-positive filopodia of U2OS cells over-expressing the MAGEA4-Cherry protein. (**D**) MAGEA4-GFP and MAGEA10-Cherry proteins were electroporated into COP5 cells and analysed 72 hours post-transfection. Analysis was performed using confocal laser scanning microscope LSM710 (Zeiss).

Next, we fused the Cherry protein to MAGEA4 and MAGEA10 C-terminus to follow the proteins in live cells. Figure 7A shows the typical cytoplasmic localization of MAGEA4-Cherry and nuclear localization of MAGEA10-Cherry proteins in U2OS cells. In the case of MAGEA4, we could sometimes follow blebs separating from the cell membrane (Figure 7A). In addition, we detected various blebs, buds and sometimes even cytoplasmic speckles containing the MAGEA4 protein (Figure 7B). The long filaments positive for the MAGEA4 protein were also observed (Figure 7C). The MAGEA10 protein localized mainly in the nucleus, but in some cells showed a weak cytoplasmic localization, however, we did not detect the formation of buds or blebs seen often with the MAGEA4 protein. In both cases, apoptotic cells, which may also be the source of EVs, were observed. Co-expression of MAGEA4-GFP and MAGEA10-Cherry proteins confirmed their different localization in the cytoplasm and nucleus of the cell, respectively (Figure 7D). Interestingly, MAGEA10 signal detected in the cytoplasm did not co-localize with MAGEA4 signal, it exhibited a pattern of intracellular vesicles (Figure 7D). In summary, we detected various budding, shedding and blebbing cells, including apoptotic cells that may produce different MAGEA-positive EVs.

## DISCUSSION

MAGE proteins have gathered interest as cancer biomarkers and immunotherapeutic targets due to their antigenic properties and unique expression pattern that is primary restricted to germ cells and aberrantly reactivated in cancers. Two decades of active research have shown their involvement in diverse cellular and developmental processes, but also raised more questions about the molecular mechanisms behind these processes. In the current study, we introduce a novel distinctive feature of MAGEA family proteins, the ability to incorporate into EVs released by cells and expose on the surface of released EVs.

We show that at least two members of MAGEA subfamily, MAGEA4 and MAGEA10, are incorporated into EVs and can be detected on the surface of EVs. This process is induced, at least to some extent, by the expression of MAGEA proteins itself. Our data show that MAGEA-EVs are rather microvesicles of different origin and/or small apoptotic bodies than classical exosomes defined by the expression of TSG101 marker protein [29]. MAGEA4 and -10 proteins are also incorporated into retrovirus MLVGag-induced virus-like particles [28] suggesting that incorporation of MAGEAs into the plasma membrane released vesicles is a more general phenomena characteristic to these proteins.

During our studies we noticed that MAGEA4 and -10 EVs have many similarities, but they also differ from each other to some extent. MAGEA family consists of 11 proteins, which share high sequence identity, but may possess different characteristics. The knowledge about the biological functions of MAGEA4 and 10 proteins is limited, however, we and others have shown that MAGEA10 is a nuclear protein while MAGEA4 is localized in the cytoplasm of the cell [5, 28]. This may explain to some extent the differences of their incorporation into EVs and may refer to different pathways for formation of MAGEA-positive EVs. We often detected stronger signal for MAGEA10 EVs in 2K fraction, which is a low speed centrifugation fraction consisting of various EVs, than in 120K fraction. This was not observed for MAGEA4 EVs. MAGEA10-EVs were also immunoprecipitated from 2K fraction with higher efficiency than from 120K (Figure 3A) and contained histones referring to small apoptotic bodies or microvesicles originating from the cell nucleus. On the other hand, we detected a lot of different MAGEA4-positive filaments and particles shed from the plasma membrane, which were not detected in MAGEA10 cells. So, it is possible that, at least to some extent, MAGEA4 and MAGEA10 EVs are of different origin and that more than one pathway is involved in formation of MAGEA-positive EVs. One possibility is, that the enhanced formation of EVs and secretion of biologically active material may be caused by senescence associated secretory phenotype (SASP), which is characteristic for cellular senescence. Accelerated cellular senescence is caused by DNA damage, reactive oxygen species (ROS) and oncogenes (OIS) recognized as a potent tumor-suppressive mechanism that arrests the growth of cells at risk for malignant transformation. However, recent studies show that senescent cells develop altered secretory activities that may induce changes in the tissue microenvironment, relaxing its control over cell behavior and promoting tumorigenesis [33]. MAGEA antigens inhibit p53 functions [14–16] and reprogram ubiquitin signaling networks to enhance DNA damage and mutagenesis tolerance in cells [34], so, it is reasonable to hypothesize that one of the mechanisms for vesiculation may be SASP.

MAGEA proteins are good immunogens which evoke a strong CTL (cytotoxic T lymphocyte) response in patients [1]. The uptake of tumor derived EVs by immune cells may have functional consequences on the immune microenvironment, which can result in either escaping the immune response or in activating immune suppression [35]. MAGEA-positive EVs may have a role in this process as microvesicles are known to modulate the immune response [36]. Secreted membrane vesicles carry both antigenic material and MHC class I molecules, that could potentially induce CD8^+^ T cell activation being a good source for antigen-presenting cells. Indeed, upregulation of MAGEA proteins by demethylating agents in osteosarcoma U2OS and HOS cells induced CTA specific CD8+ T-cell responses and facilitated tumor cell killing *in vivo* [32]. Our study of the antibody response against MAGEA4/10 proteins in patients with melanoma at different stages of disease revealed highest number of strongly responding patients among stage II, but no response in stage IV patients [37], which allows us to suggest that MAGEA-EVs may have a role in modulation of immune response.

The function of microvesicles is dependent on the cargo they carry. Once shed, microvesicles can cover some distance, thus enabling the horizontal transfer of bioactive molecules and deposition of backed bioactive effectors at distal sites. Cargo contained within microvesicles may be released into the extracellular milieu with consequences for the surrounding environment [38]. Cancer cells have been found to release higher quantities of EVs compared to normal cells and these EVs contain oncogenic compounds which may trigger various signaling pathways in recipient cells once incorporated [38, 39]. What is the potential biological role of MAGE-EVs? MAGE-A proteins are associated with poorer outcomes for the patients [10, 11] and one possibility is that cancerous cells expressing MAGEA proteins secret EVs that could migrate through the bloodstream and influence new cells to become cancerous. EVs may also help to establish microenvironment and a nische for cancer growth. MAGEA3/6 expression has shown to drive several hallmarks of cancer such as cell proliferation, cell migration, invasion and anchorage-independent growth and are also sufficient to drive tumorigenic properties of non-cancerous cells [22]. Liu *et al*. have demonstrated that the expression of MAGEA3 promoted tumor cell migration and enhanced invasive cancer cell growth *in vitro* and *in vivo* [17].

Cancer cells secrete more EVs than normal, and consequently, patients with cancer have higher levels of EVs compared to healthy individuals [40]. So, the increased level of EVs in circulation and the packaging of cancer related molecules may serve as promising biomarkers for cancer diagnosis [39, 41]. However, despite the great promise of EV-based diagnosis, efficient capture of cancer cell-derived EVs apart from normal cell-derived EVs is still challenging [39]. The other possibility is to use cancer-derived EVs as direct targets for the treatment of cancer. Currently there are a lot of EV-inhibiting reagents available [39], but these reagents also induce off-target changes as many of them are major regulators of cell functions. More of investigations are needed to find and test specific tools against cancer-related EVs and the deep characterization of MAGEA-positive EVs may add one piece to the list of potential cancer-related targets.

## MATERIALS AND METHODS

### Cells, plasmids and transfection

Mouse fibroblast cells Cop5 [28] and human osteosarcoma cells U2OS, which were obtained from the American Type Culture Collection (ATCC no: HTB-96) were cultured in IMDM medium supplemented with 10% fetal calf serum, penicillin (100 U/ml), and streptomycin (100 ng/ml) at 37° C. Cop5 cells were transfected with expression plasmids pQM-MAGEA4, pQM-MAGEA10 or with plasmid containing truncated MAGEA4 proteins pQM-MAGEA4-105 and pQM-MAGEA4-161, then cultured in IMDM medium supplemented with 5% exosome free fetal calf serum, penicillin (100 U/ml), and streptomycin (100 ng/ml). As a negative control, plasmids without the MAGEA insert were used. Transfection was carried out at 230V and 975μF on the BioRad GenePulser Xcell^TM^ device as described in [28].

Plasmids pQM-MAGEA4 and pQM-MAGEA10 express the MAGEA coding sequences fused in-frame with C-terminal E2Tag epitope under the control of CMV promoter [28]. The truncated MAGEA4 proteins were generated by PCR and cloned into pQM-CMV-E2Tag/C vector (Icosagen). pQM-Cherry-MAGEA4 and pQM-Cherry-MAGEA10 constructs were made by cloning the MAGEA coding sequences into pQM-CTag-mCherry vector [30]. For MAGEA4-GFP, MAGE4 coding region was cloned into pEGFP-N1 vector in frame with EFGP-coding sequence. All the sequences were verified by sequencing.

### Purification of EVs

Isolation of EVs was carried out as described in [29]. The cell culture media was collected 72 hours after transfection, and centrifuged as described. The first centrifugation at 300 g 10 min was carried out to remove dead cells and cell debris. Then the supernatant was centrifuged at 2000 g 20 min to precipitate apoptotic bodies and other vesicles of similar size (2K fraction). The next centrifugation was carried out at 16500 g for 20 min (16K fraction) and the third at 120 000 g (27 000 rpm; Beckman Coulter OptimaTM L-90K Ultracentrifuge, rotor SW28) for 70 min to precipitate small EVs (120K fraction). The pellets were suspended in 200 μl of PBS. Washing with PBS included centrifuging of EVs from the 2K and 16K fractions at 17000 g for 15 minutes at 4° C using the MicroCL 21R Centrifuge (Thermo Scientific) and EVs of the 120K fraction at 32000 rpm for 1.5 hours at 4° C using the Beckman Coulter OptimaTM L-90K Ultracentrifuge, rotor SW55. The final EVs were resuspended in 100 μl of PBS. The stepwise sucrose density gradient centrifugation was performed as depicted in Kurg *et al* [28] with loading the probe either on the top or bottom of the tube, respectively. The protein concentrations were measured with the Bradford Protein Assay using BSA as a standard (Bio-Rad Laboratories; USA). In average, we obtained approximately 35 μg of 2K, 10 μg of 16K and 50 μg of 120K from 4 × 10^7^ of transfected cells. Students paired *t*-test with a two-tailed distribution was used to calculate *p*-values.

### Western blot and immunoprecipitation analysis

Expression of the MAGE-A proteins in both cells and EVs was detected using the Western blot analysis. Protein samples were suspended in Laemmli buffer and denatured for 10 min at 100° C. 5 μl of cell lysates and 10 μl of EV lysates per lane were separated electrophoretically using 10% SDS-PAGE gel and blotted onto a PVDF membrane using Trans-Blot SD Semi-Dry Transfer Cell (BioRad). Affinity-purified rabbit polyclonal antibodies against MAGEA4 (2.5 mg/ml) and MAGEA10 (1.1 mg/ml) [28] were used for immunoblotting at dilutions of 1:25000 and 1:2200, respectively. Alpha-tubulin (dilution 1:4000; T5168; Sigma-Aldrich), anti-TSG101 (dilution 1:10000, T5701; Sigma), anti-flotillin-1 (dilution 1:1000; 610820; BD Transduction Laboratories), anti-integrin beta 1 (dilution 1:2000; ab179471; Abcam), anti-histone H3 (dilution 1:1000; ab1791; Abcam), Mab 6C1 (sc-20034; Santa Cruz) and anti-E2Tag antibody 5E11 (dilution 1:10000; Icosagen) were used in different experiments. Goat anti-rabbit (1 mg/ml, LabAS) and goat anti-mouse (1 mg/ml, LabAS) antibodies were used as secondary antibodies at a dilution of 1:10000. Protein signals were detected using ECL Western blotting (GE Healthcare) reagents. The staining of SDS-PAGE gels was performed with PageBlue Protein Staining Solution (Thermo Scientific).

Immunoprecipitation was performed with 20 μg of EVs in 200 μl of binding buffer containing 0.1% Tween20, 10% glycerol, 0.5 mM DTT and proteasome inhibitors in PBS. Anti-E2Tag specific antibody 3F12 (1 μg) (Icosagen Ltd.) or anti-MAGEA4/MAGEA10 antibodies [28] were added and incubated 1 hour at 4° C using end-over-end rotation. After addition of 5 μl of protein G magnetic beads (Dynabeads) and further incubation for 1 hour at 4° C, the magnetic beads were washed four times with 200 μl of binding buffer, resuspended in 20 μl of Laemmli buffer and analyzed by Western blotting.

### Flow cytometry

Flow cytometry analyses were conducted to analyze the surface expression of MAGEA proteins in different EVs. 10 μg of EVs from each sample were incubated with 10 μl of 4 μm diameter aldehyde/sulphate latex beads (Molecular Probes; Life Technologies) for 15 minutes at room temperature. Then PBS was added to a final volume of 1 ml and the mix was incubated at 4° C overnight using end-over-end rotation. The beads were then blocked with a 100 mM glycine/PBS solution for 30 minutes at room temperature and washed twice with 0.5% BSA in PBS. Incubation with affinity-purified rabbit polyclonal antibodies against MAGE-A4 (final concentration of 1 ng/μl) or MAGE-A10 (2 ng/μl)) [28] or mouse monoclonal antibodies 6C1 (sc-20034; Santa Cruz) and anti-E2Tag 3F12 (Icosagen) were carried out in 0.5% BSA in PBS for 1 hour at 4° C using end-over-end rotation. The samples were then washed twice. Anti-rabbit or anti-mouse Alexa 488 antibodies (1 mg/ml, dilution 1:1000, Invitrogen) were used as secondary antibodies and samples were incubated with it for 1 hour at 4° C using end-over-end rotation. The beads were washed twice, resuspended in 300 μl of 0.5% BSA in PBS and analyzed with the LSR II device (BD Biosciences) using the BD FACSDiva Software (BD Biosciences). Analysis of the results was carried out with the FlowJo VX program (Tree Star).

### Confocal microscopy

Indirect immunofluorescence analysis was performed as described in [28] with U2OS cells or with U2OS cells transfected by electroporation with pQM-MAGEA4 plasmid. Antibodies against MAGEA4 (final 2 μg/ml) [28], β-actin (A2228; Sigma-Aldrich; dilution 1:200) and α-tubulin (T5168; Sigma-Aldrich; dilution 1:1000), and secondary antibodies conjugated with Alexa-488 and Alexa-568 (Invitrogen) were used. For live cell images, U2OS cells transfected with pQM-Cherry-MAGEA4 and pQM-Cherry-MAGEA10 plasmids, were grown in 8-well chambered coverglass (Lab-Tek^®^, Nunc^®^; Thermo Scientific) and analyzed 24 or 48 hours after transfection. Hoechst 33342 (final concentration 2.5 μg/ml) was added 15 minutes before microscope analysis to label the nuclei. Analysis was performed using confocal laser scanning microscope LSM710 (Zeiss). Images were obtained with 63x lens and analyzed by ZEN2011 software.

### Analysis of EVs by DLS and NTA.

DLS (Dynamic Light Scattering) measurements were performed with Zetasizer Nano (Malvern Instruments; UK) as described in [28]. NTA (Nanoparticle tracking analysis) was performed with a ZetaView nanoparticle analyser (Particle Metrix GmbH; Germany). Before each session, the size and concentration of standard silica beads was measured. In all cases, 11 measurements were recorded in at least one dilution and analyzed using the ZetaView Software 7.11 with default settings. Graphics and statistical analysis were done with Excel software. Students paired *t*-test with a two-tailed distribution was used to calculate *p*-values.
